# Species Composition, Seasonal Abundance, and Biting Behavior of Malaria Vectors in Rural Conhane Village, Southern Mozambique

**DOI:** 10.3390/ijerph20043597

**Published:** 2023-02-17

**Authors:** Graça Salomé, Megan Riddin, Leo Braack

**Affiliations:** 1 UP Institute for Sustainable Malaria Control, School of Health Systems and Public Health, Faculty of Health Sciences, University of Pretoria, Pretoria 0028, South Africa; 2 Department of Physiological Sciences, Faculty of Medicine, Eduardo Mondlane University, 702 Salvador Allende Ave., Maputo P.O. Box 257, Mozambique; 3 Malaria Consortium, Faculty of Tropical Medicine, Mahidol University, 420/6 Rajavithi Rd, Ratchathewi, Bangkok 10400, Thailand

**Keywords:** *Anopheles*, feeding behavior, Mozambique, seasonal abundance

## Abstract

Malaria vector surveillance provides important data to inform the effective planning of vector control interventions at a local level. The aim of this study was to determine the species diversity and abundance, biting activity, and *Plasmodium* infectivity of *Anopheles* mosquitoes from a rural village in southern Mozambique. Human landing catches were performed monthly between December 2020 and August 2021. All collected *Anopheles* were identified to the species level and tested for the presence of malaria parasites. Eight *Anopheles* species were identified among the 1802 collected anophelines. *Anopheles gambiae* sensu lato (s.l.) were the most abundant (51.9%) and were represented by *Anopheles quadriannulatus* and *Anopheles arabiensis*. *Anopheles funestus* s.l. represented 4.5%. The biting activity of *An. arabiensis* was more pronounced early in the evening and outdoors, whereas that of *An. funestus* sensu stricto (s.s.) was more intense late in the night, with no significant differences in location. One *An. funestus s.s.* and one *An. arabiensis*, both collected outdoors, were infected with *Plasmodium falciparum*. The overall entomologic inoculation rate was estimated at 0.015 infective bites per person per night. The significant outdoor and early evening biting activity of *An. arabiensis* and *An*. *funestus* found in this village may negatively impact the effectiveness of current vector control interventions. Additional vector control tools that can target these mosquitoes are needed.

## 1. Introduction

Malaria vector control interventions for large-scale deployment mainly rely on the use of insecticide-treated nets (ITNs) and indoor residual spraying (IRS) [1], and these interventions target indoor biting and resting mosquito vectors [2]. The scale-up of long-lasting insecticidal nets (LLINs) in sub-Saharan Africa led to an increase in the coverage from 5% in 2000 to 68% in 2021 [3]. The deployment of ITNs and IRS interventions in this region have contributed significantly to the reduction in malaria transmission and mortality between 2000 and 2015 [4]. However, the burden of malaria is still high in the World Health Organization (WHO) African region, where 95% of the 247 million cases in the world and approximately 96% of the 619,000 deaths occurred in 2021 [3]. To understand the impact of vector control interventions on malaria transmission and mortality, it is important to understand human and vector behavior, as it may influence the effectiveness of these interventions [5,6,7]. Entomological surveillance, an important and essential activity, provides data on vector composition, distribution, seasonal abundance, biting and resting behavior, rates of infection with *Plasmodium*, susceptibility to insecticides, and breeding sites [8]. The knowledge of these vector characteristics is important for planning and selecting vector control interventions at a local level [8].

In Africa, the primary malaria vectors belong to the *Anopheles gambiae* s.l. and *Anopheles funestus* s.l. [9]. *Anopheles gambiae* s.s. and *An. funestus* s.s. populations have been reduced by means of the application of vector control interventions in some settings in Africa [10,11,12], as these species are strongly anthropophilic and endophilic [13]. Changes in species composition and feeding behavior have been reported after the implementation of vector control interventions, including LLINs and IRS [11,14]. Such changes may reflect the behavior plasticity of some *Anopheles* species that search for alternative hosts, as observed with *An. arabiensis* [5,15], or biting early in the evening [16] or late in the morning, as observed in *An. funestus* s.s. [17]. This plasticity in behavior and the reduced susceptibility to insecticides applied to bed nets and walls indoors [18,19] impacts the effectiveness of IRS and LLINs and can at least partly explain the residual transmission of malaria [2,20]. The increasing proportions of outdoor biting [11,21] and of the activity of secondary vectors [22,23,24] contribute to residual transmission with implications in malaria epidemiology. To address these challenges, it is important to improve the existing vector control tools or develop new complementary tools that can target these exophilic and exophagic vectors.

Currently favored complementary vector control interventions include larval source management and house screening [1]. These complementary measures have proven to be effective [25,26], but the application of larval source management is limited to areas where the breeding sites are “few”, “fixed”, and “findable”, while house screening depends on community affordability, acceptance, and compliance [1]. Topical repellents can reduce exposure to mosquitoes outdoors [27]; however, their impact on reducing malaria transmission at the population level has not been demonstrated and is thus not endorsed by the WHO [28]. Therefore, further research of repellent-based tools that are affordable and long-lasting and that can target outdoor biting mosquitoes, especially in rural areas where the malaria burden is high [29], is required.

Developing, testing, and deploying vector control tools need to be driven by data on local vector bionomics. Repellent-impregnated strands and socks were developed as part of a multilateral research project at University of Pretoria—Institute of Applied Material [30,31]. Using procedures such as those described by Sibanda et al. (2018) [30], footwear was developed to be a potential new complementary vector control tool (Sibanda et al., *pers. comm.*). The efficacy tests of this tool were planned to be performed in Conhane, a rural Village in southern Mozambique. Currently, however, there are no available data on the composition, abundance, and biting behavior of malaria vectors present in the village selected despite being situated in an area with an irrigation scheme that provides permanent mosquito breeding sites.

The objectives of this study were (i) to determine the species composition and seasonal abundance of host-seeking mosquitoes in Conhane, a rural village in southern Mozambique; (ii) to describe the biting activity and preferred biting location of host-seeking mosquitoes in Conhane Village, southern Mozambique; and (iii) to determine sporozoite rate and entomologic inoculation rate of host-seeking *Anopheles* mosquitoes in Conhane Village southern Mozambique.

## 2. Materials and Methods

### 2.1. Study Area

This study was conducted in Conhane (24°41′ S, 33°06′ E), a village in Chókwè District, in the southern part of Mozambique, from December 2020 to August 2021. Chókwè, a region that spans an area of 2466 square kilometers with an estimated population of 232,196 inhabitants in 2021, is situated in the Limpopo River floodplain savannah [32,33]. It is bordered in the north by Guija and Mabalane Districts (separated by Limpopo River), in the northwest by Massingir District, in the southwest by Magude District, in the southeast by Bilene District, and in the east by Chibuto District [32]. The climate of Chókwè is tropical dry with two seasons: one hot and rainy that occurs from October to March and one cold and dry from April to September. The vegetation is mainly dry savannah composed of grass with moderate density of *Acacia* spp. and *Combretum* spp. trees [34]. The average annual rainfall is between 500 and 800 mm, with rainfall occurring during the summer season. Average annual temperatures range between 22 and 26 °C with relative humidity (RH) between 60 and 65% and evapotranspiration of 1500 mm [31,34].

**Figure 1:**
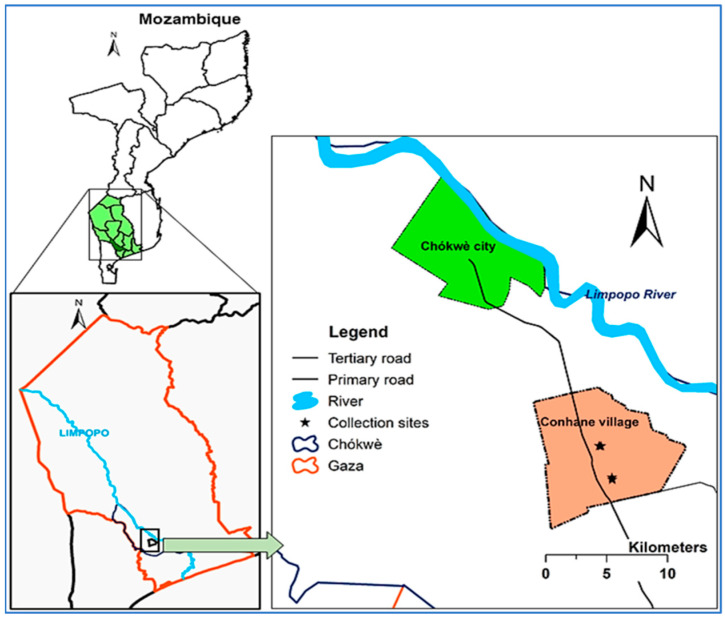
Map showing Conhane Village in Chókwè, Gaza Province, Mozambique.

Conhane Village is located 25 km southeast of Chókwè City (Figure 1) and is situated in the Chókwè irrigation scheme. The village is divided into three distinct clusters of households by one main and one secondary irrigation canal. Two of these clusters comprise seven small neighborhoods that are contiguous and are collectively named Conhane neighborhoods. To differentiate the village from the neighborhoods, which share the same name, hereafter, the village is referred to as Conhane Village and the Conhane neighborhoods are referred to as Conhane. The third cluster is composed of one neighborhood, which is considered the primary neighborhood, and is named Cotsuane. Conhane and Cotsuane are separated by 1 km at their closest edges. Socioeconomic demographic factors include a dominancy of community members with subsistence livelihoods, with the primary livelihood being agriculture [32]. Domestic livestock (cattle and goats) rearing and husbandry is practiced on a small scale, and kraals are predominantly built and maintained near to the households. Houses are constructed with mud and reeds/sticks/stone, or block and cement walls, with thatched, corrugated iron, or tile roofs [32].

Malaria is endemic in Chókwè, with intense transmission occurring during the rainy season when the full population is at risk. Conhane Village has an estimated population of 5150 inhabitants and is the village in the Chókwè Health and Demographic Surveillance System (HDSS) area that reported the most cases of malaria in 2018 (Bonzela et al., *pers. comm.*). The marshes found at the edges of the village and the irrigation scheme that provides jobs for a portion of local people are known to produce mosquito larval breeding sites all year, thus influencing malaria epidemiology in the study region. These characteristics and accessibility during the study period influenced the choice of this village for this study.

### 2.2. Meteorological Data

Rainfall, average temperature, and relative humidity data from Chókwè were obtained from National Meteorological Institute in Maputo, Mozambique.

### 2.3. Mosquito Collection

Ten volunteers from the community, six females and four males of ages ranging between 19 and 50, were selected and trained to collect mosquitoes from the collection sites, Conhane and Cotsuane. Host-seeking mosquitoes were collected from December 2020 to August 2021 using the human landing catch (HLC) technique, as described by Gimnig et al. [35], which is considered the gold standard for the monitoring of malaria vector populations. Two houses near a permanent mosquito breeding site were selected in each site with the distance between the houses being between 100 and 200 m. Mosquito collections were performed monthly on two consecutive nights. Each night two volunteers per house collected mosquitoes in hourly sessions from 18:00 to 06:00 a.m. Each collection session lasted 45 min with a 15 min comfort break. One volunteer was positioned indoors and the other outdoors, 10 m from the house. Volunteers changed positions in the house each night and rotated through the houses each month to account for individual variability of attractiveness and collection skills. The volunteers were seated on plastic chairs with exposed legs and feet. Each volunteer was provided with a flashlight and a polystyrene cup (one per 45 min session). As mosquitoes began to probe the exposed legs and feet, the volunteer aspirated using a mouth aspirator and then transferred them into a labeled polystyrene cup.

Mosquitoes collected each hour were stored temporarily in polystyrene cups covered with mesh and labelled with the sampling site, volunteer and house codes, and hour of collection. During the night, the cups with collected mosquitoes were kept in a cool polystyrene box, each cup with a wad of cotton wool moistened with 10% sugar solution; these cups were then transported to the laboratory of the Chókwè Health Research and Training Centre (Centro de Investigação e Treino em Saúde de Chókwè—CITSC) the next morning. In April 2021, collections were performed only on one night due to logistic and weather constraints.

### 2.4. Laboratory Processing

#### 2.4.1. Morphological Identification

After each night of collection, all mosquitoes were euthanized by freezing at –20 °C for a minimum of one hour. Individuals were then sorted into genera and counted. All the mosquitoes from the *Anopheles* genus were identified morphologically to the species level where possible, or species complex or group, using a stereomicroscope and dichotomous keys [13,36]. Mosquitoes were stored individually in Eppendorf tubes with silica gel and labelled with species, date, collection site, hour, and location of collection and were kept at –20 °C until transportation to National Institute of Health (Instituto Nacional de Saúde—INS) for further laboratory analyses.

#### 2.4.2. Molecular Identification

Mosquitoes from the morphologically indistinct *Anopheles funestus* s.l. and *An. gambiae* s.l. species were further molecularly identified to the species level using PCR assays. DNA extracted from the wings and legs was amplified by means of PCR as previously described [37,38].

#### 2.4.3. Circumsporozoite Protein Detection

A sandwich enzyme-linked immunosorbent assay (ELISA) was performed to detect circumsporozoite protein (CSP) of *Plasmodium falciparum*, *P. vivax* 210 and *P. vivax* 247 [39,40,41]. The head and thorax of *An. funestus* s.l., *An. gambiae* s.l., *An. pharoensis*, *An. squamosus*, *An. tenebrosus, An. ziemanni*, and *An. coustani* mosquitoes were used. These mosquito species have been previously incriminated as either primary or secondary malaria vectors [23,42,43,44,45].

### 2.5. Data Analysis

All collected anopheline mosquitoes were sorted by genus, site, and location (indoor or outdoor). *Anopheles* mosquito composition was expressed as the proportion of each species for the study period. The seasonal abundance and distribution of *Anopheles* species was expressed as frequencies and proportions. Differences in composition and seasonal abundance of mosquitoes between sites and location were tested using chi-squared tests. When post hoc analysis was necessary, it involved pairwise comparisons of two proportions using multiple chi-squared tests for homogeneity with Bonferroni correction (significance accepted at *p <* 0.0167). The human biting rate (HBR) was expressed per species as the number of collected mosquitoes per person per night (bpn). The HBR of the *Anopheles* mosquito species was compared by location and study site using the Kruskal–Wallis H-test or Mann–Whitney U-test, as the distribution was not normal. When multiple comparisons were performed, Bonferroni corrections were conducted, and the adjusted *p*-values were reported. The sporozoite rate (SR) was determined by dividing the number of infected mosquitoes by the total number of analyzed mosquitoes. The entomologic inoculation rate (EIR) was obtained by multiplying the SR by the HBR [46]. All the analyses were conducted considering 95% confidence levels and 5% of significance. Data were analyzed using IBM SPSS for Windows, version 28.0.1.0(142) (IBM Corp, Armonk, NY, USA).

## 3. Results

A total of 13,059 host-seeking mosquitoes were collected in Conhane Village over 136 person-nights from December 2020 to August 2021. The distribution of collected mosquito genera by site is shown in Table 1. The most prevalent genera were *Mansonia* (46.9%), *Culex* (27.7%), and *Anopheles* (13.8%), with the least represented being *Aedes* (0.5%). Overall, more mosquitoes were collected in Cotsuane (65.5%) than in Conhane (34.5%), and the difference in the distribution of all mosquitoes between the sites was statistically significant (χ^2^ = 191.762, *df* = 1, *p* < 0.001).

### 3.1. Composition of Anopheles Species in Conhane Village

A total of 1802 female *Anopheles* mosquitoes belonging to eight species or morphologically indistinct species complexes or groups were collected. Of these, 51.9% were *An. gambiae* s.l. and 4.5% were *An. funestus* s.l. The other anophelines comprised *Anopheles tenebrosus* (25.1%), *Anopheles ziemanni* (10.9%), *Anopheles pharoensis* (4.3%), *Anopheles squamosus* (2.2%), and *Anopheles coustani* s.l. (0.9%). Only *An. funestus* s.l. and *An. gambiae* s.l. were further considered in the analysis.

*Anopheles funestus* s.l. and *An. gambiae* s.l. mosquitoes were tested with PCR to identify the sibling species. Six mosquitoes identified morphologically as being *Anopheles parensis* were not considered for PCR due to having already been identified morphologically. All 76 *An. funestus* s.l. were confirmed as being *An. funestus* s.s. Of the 936 *An. gambiae* s.l. tested with PCR, 812 (86.8%) were identified as *Anopheles quadriannulatus* and 117 (12.5%) as *Anopheles arabiensis*, while 7 (0.7%) did not amplify.

Table 2 shows the distribution of *An. funestus* s.s., *An. arabiensis*, and *An. quadriannulatus* by site. All these species were present in both sites. The overall difference in the proportions of mosquito species between the sites was statistically significant (χ^2^ = 27.34, *df* = 2, *p* < 0.001). Post hoc analysis revealed that the differing proportions of these mosquito species between the two sites was statistically significant for *An. arabiensis* and *An. funestus* s.s. (χ^2^ = 14.59, *df* = 1, *p* < 0.001 and χ^2^ = 15.59, *df* = 1, *p* < 0.001, respectively) but not for *An. quadriannulatus* (χ^2^ = 0.42, *df* = 1, *p* = 0.52).

The indoor and outdoor composition and proportions of the *Anopheles* species are shown in Table 3. All three species were collected both indoors and outdoors. Although the overall variation between indoor and outdoor proportions of these *Anopheles* species was statistically significant (χ^2^ = 33.49, *df* = 1, *p* < 0.001), post hoc analysis revealed that the variation was significant for *An. funestus* s.s. and *An. quadriannulatus* (χ^2^ = 31.84, *df* = 1, *p* < 0.001, and χ^2^ = 18.94, *df* = 1, *p* < 0.001 respectively) but not significant for *An. arabiensis* (χ^2^ = 0.48, *df* = 1, *p* = 0.53).

### 3.2. Rainfall and Seasonal Abundance of Mosquitoes

**Figure 2:**
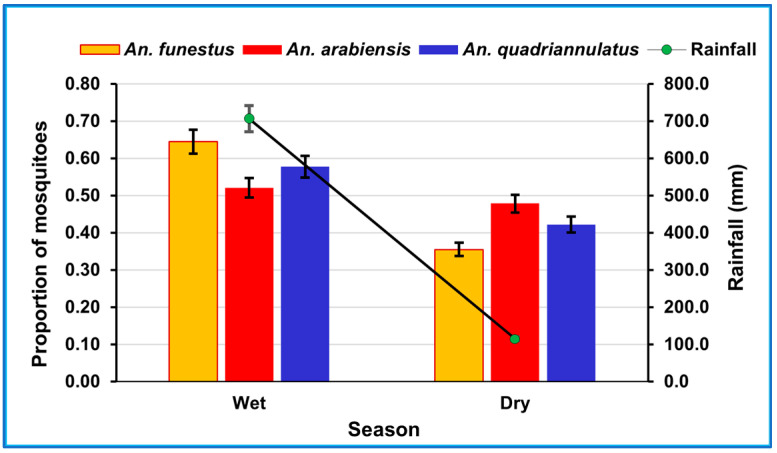
Proportions of *An. funestus* s.s. (n = 76), *An. arabiensis* (n = 117), *An. quadriannulatus* (n = 812), and rainfall (total = 822 mm) by season in Conhane Village.

The cumulative rainfall over the study period was 822 mm. Approximately 86% of this rain fell in the period from December to March and 14% in the period from April to August. The proportions of *An. funestus s.s.*, *An. arabiensis*, and *An. quadriannulatus* were slightly higher in the wet season than in the dry season (Figure 2). However, the differences in the proportions of these mosquito species between the seasons were not statistically significant (χ^2^ = 2.91, *df* = 2, *p* = 0.23).

### 3.3. Human Biting Rates

The combined HBR of *An. quadriannulatus*, *An. arabiensis*, and *An. funestus* was 7.4 bpn. *Anopheles quadriannulatus* had the highest biting rate (6.0 bpn), followed by *An. arabiensis* (0.9 bpn) and *An. funestus* s.s. (0.6 bpn).

**Figure 3:**
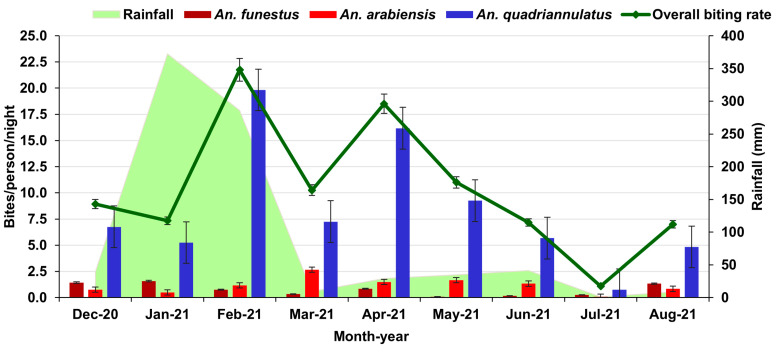
Monthly human biting rates of *An. funestus* s.s., *An. arabiensis*, and *An. quadriannulatus*, and rainfall in Conhane village.

The monthly HBRs of *An. funestus* s.s. and *An. arabiensis* remained relatively constant over the study period. The *An. funestus* s.s. peak was in January (1.2 bpn), while *An. arabiensis* peaked in March (2.0 bpn) (Figure 3). The monthly HBR of *An. quadriannulatus* was substantially higher than those of *An. funestus* s.s. and *An. arabiensis* and exhibited two peaks, one in February (14.9 bpn) and the other in April (12.1 bpn) (Figure 3). This was followed by a gradual decline to a minimum in July (0.6 bpn).

The overall HBR in Conhane (4.2 bpn) was lower than that in Cotsuane (10.6 bpn), and the difference in the respective mean ranks was statistically significant (*U* = 3063, *z* = 3.287, *p* = 0.001). The *Anopheles funestus* HBR was higher in Cotsuane than that in Conhane (*U* = 3146.5, *z* = 4.789, *p* < 0.001), whereas the *An*. *quadriannulatus* HBR was higher in Conhane than that in Cotsuane (*U* = 1584.5, *z* = –3.205, *p* = 0.001). The *Anopheles arabiensis* HBR biting rate was similar in both sites (*U* = 2607.5, *z* = 1.478, *p* = 0.139).

*Anopheles funestus* s.s. was found to be biting equally indoors and outdoors (*U* = 2118.0, *z* = –1.113, *p* = 0.266). However, the outdoor HBRs were higher than the indoor ones for *An*. *arabiensis* (*U* = 2983, *z* = 3.357, *p* < 0.001) and *An*. *quadriannulatus* (*U* = 3566.0, *z* = 5.525, *p* < 0.001).

### 3.4. Night-Biting Cycle

**Figure 4:**
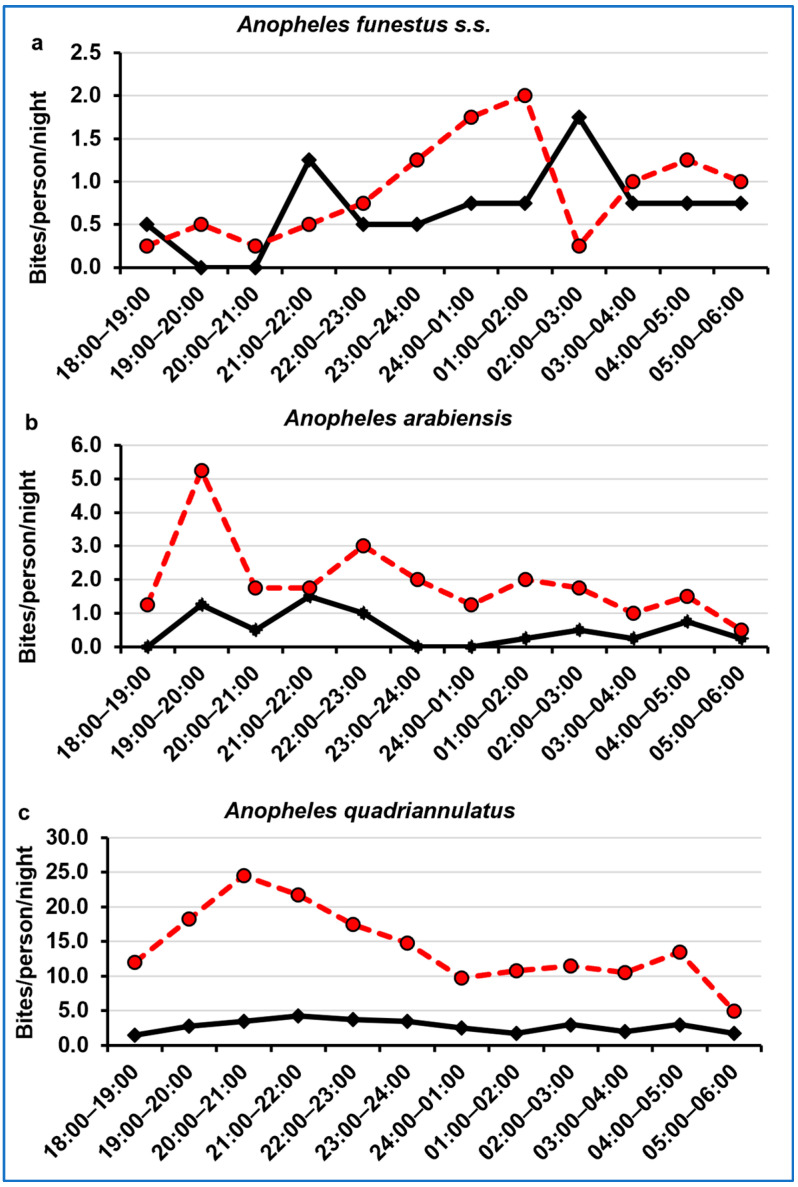
Hourly night human biting rates of *An. funestus* s.s. (**a**), *An. arabiensis* (**b**), and *An. quadriannulatus* (**c**) from Conhane village. The continuous lines represent the biting rates indoors, and the dashed lines represent the biting rates outdoors.

The *An. funestus s.s.* indoor and outdoor night biting activity varied throughout the night, particularly with regards to location, with the highest intensity (Figure 4a). The biting activity started to increase gradually from 21:00 p.m., peaked between 01:00 a.m. and 02:00 a.m. outdoors and between 02:00 a.m. and 03:00 a.m. indoors, and then decreased slightly until 05:00 a.m. For *An. arabiensis,* the biting activity was higher outdoors than indoors and was higher in the first half of the night (18:00 p.m.–24:00 p.m.) than in the second half (24:00 p.m.–06:00 a.m.) (Figure 4b). The peak of biting activity was reached between 19:00 p.m. and 20:00 p.m. outdoors and between 21:00 p.m. and 22:00 p.m. indoors. The *An. quadriannulatus* indoor biting activity was lower than that outdoors (Figure 4c). The biting activity was more intense in the first half of the night, reaching a peak between 20:00 p.m. and 21:00 p.m. indoors and between 21:00 p.m. and 22:00 p.m. outdoors, finally decreasing gradually until 05:00 a.m.

### 3.5. Sporozoite Rate and Entomologic Inoculation Rate

A total of 1789 female anophelines were tested for the presence of *Plasmodium. falciparum*, *P. vivax* 210 and *P. vivax* 247 CSP. This included 936 *An. gambiae* s.l. (812 *An. quadriannulatus*, 117 *An. arabiensis,* and 7 *An. gambiae* s.l. that did not amplify on PCR analyses), 76 *An. funestus* s.s., 452 *An. tenebrosus*, 197 *An. ziemanni*, 73 *An. pharoensis*, 36 *An. squamosus*, 16 *An. coustani*, and 3 *An. parensis* individuals.

From the tested mosquitoes, two were found to be positive for *P. falciparum* CSP presence: one *An. funestus* s.s. and one *An. arabiensis*. This resulted in an overall SR for these two species (n = 193) of 1.04%. *Anopheles funestus* s.s. was the species with the highest SR (1.32% 1/76), followed by *An. arabiensis* (0.85% 1/117). All the positive mosquitoes were collected outdoors. The infected *An. arabiensis* was collected in March 2021 between 19:00 p.m. and 20:00 p.m. in Conhane, and the infected *An. funestus* s.s. was collected in August 2021 between 01:00 a.m. and 02:00 a.m. in Cotsuane. The overall EIR for the two species was 0.015 infective bites per person per night (with 0.0074 infective bites per person per night for each species).

## 4. Discussion

This study is the first report of mosquito composition, abundance, and biting behavior of malaria vectors from Conhane Village in Chókwè District, southern Mozambique. Understanding the composition of mosquito species, ecology, and biting behavior is integral to the selection of appropriate interventions and deployment in time and space towards effective vector control [47,48]. This study provides an understanding of the *Anopheles* species diversity, seasonal abundance, and biting and sporozoite rates in a highly understudied malaria burden region.

Over the collection period, eight species of *Anopheles* mosquitoes were collected. *Anopheles. gambiae* s.l. and *An. funestus* s.l. were predominantly collected, which include members recognized as major malaria vectors, with reported sporozoite rates varying across Africa. In Mozambique, *An. funestus s.s., An. arabiensis*, and *An. gambiae s.s.* are the main implicated vectors, while *An. merus* is implicated in some localities [22,49,50].

*Anopheles gambiae* s.l. mosquitoes were the most predominant among the collected anophelines, similar to findings reported in previous studies elsewhere in southern Africa [6,49,51]. PCR identification of *An. gambiae* s.l. revealed the presence of *An. arabiensis* and *An. quadriannulatus*, with no detection of *An. gambiae* s.s. This distribution of *An. gambiae* s.l. species differs from reports from the central province of Zambezia in Mozambique, where s.l. was only represented by *An. gambiae* s.s. and *An. arabiensis* [49,52]. *Anopheles quadriannulatus* distribution is within eastern and southern Africa, often in sympatry with *An. arabiensis*, with previous records in South Africa, Mozambique, Malawi, Zimbabwe, Zambia, and Botswana [47,53,54,55,56,57,58,59]. Studies conducted in other villages in Chókwè reported the occurrence of mosquitoes from *An. gambiae* s.l. but in lower proportion than *An. funestus* s.l. [53,59,60]. Casimiro (2003) did not report the presence of *An. funestus* s.s. but reported relatively high proportions of *An. arabiensis* when compared with *An. quadriannulatus* resting indoors in Chókwè [61]. Differences in the sampling techniques may explain the varied composition and abundance of these species observed in the Chókwè region. *Anopheles funestus* s.s. has a wide distribution in western, eastern, and southern Africa [13,62], and in Mozambique, the distribution of *An. funestus* s.s. is widespread across the country [53,63,64].

Based on abundance, the predominant malaria vector species were identified as *An. arabiensis and An. funestus* s.s. These two species of mosquito showed differences in distribution, biting rates, and location (outdoors versus indoors), but with no significant seasonal variations.

*Anopheles funestus* was significantly more abundant in Cotsuane, while *An*. *arabiensis* was more abundant in Conhane. Factors such as the temperature, rainfall, vegetation, and land usage influence the abundance and distribution of mosquitoes [65,66,67,68]. Different bioecological areas can exhibit differences in the macro- and microenvironment, which can result in unequal distribution of mosquito species [69]. Conhane and Cotsuane are situated in an irrigation scheme that provide permanent breeding sites. These results could be explained by differences in the distribution of preferred breeding sites of the two *Anopheles* species between Cotsuane and Conhane [70,71,72]

In the present study, *An. arabiensis* was found to be biting more frequently outdoors than indoors. Similar behavior was reported in Ethiopia and other parts of Mozambique [15,73]. This species exhibits a wide range of feeding behavioral patterns in varying situations [74]. *Anopheles* arabiensis have been found to be exophagic, endophagic [75], or both [76], with dependence on host availability, ecological conditions, and implementation of indoor vector control interventions. *Anopheles quadriannulatus* is often described as a zoophilic and exophagic vector [74,77], although plasticity with respect to host choice has been reported in experimental studies [78,79]. High proportions of host-seeking *An. quadriannulatus* have been found in the present study, and the same finding was previously reported in Zambia [56]. *Anopheles funestus* s.s. is an archetypal endophagic mosquito [80], but contrasting feeding behavior in this species has been reported in different parts of Africa, with reports of populations being more endophagic [50,73,81], more exophagic [16,82], or both [56,76,83]. Within this study, *An. funestus* did not show preference for indoor or outdoor biting.

Precipitation and temperature are factors that influence the presence and abundance of mosquito vectors, as these factors drive the availability of suitable breeding sites and the duration of the gonotrophic cycle [65,66,67]. In general, present populations of *An. gambiae* s.l. and *An. funestus* s.l. start to increase at the beginning of the rainy season, with *An. gambiae* s.l. attaining a mid-season peak and *An. funestus* s.l. peaking at the end of the season, with both decreasing in the dry season [84,85,86,87]. The proportion of rainfall over the period of the present study was significantly higher in the wet season. This finding was not accompanied by a significant proportional increase in the populations of *An. funestus* s.s., *An. quadriannulatus*, and *An. arabiensis*. The persistence of *An. arabiensis and An. funestus* s.s. populations during the dry season has been highly reported in Africa [55,88,89]. This dry season persistence phenomenon may be possible within this study region due to the availability of permanent ditches and small ponds created by the water accumulated after rainfall, allied with the presence of irrigation scheme canals and rice fields [90,91]. In the study region, malaria cases are reported all year round (NMCP-Chókwè focal point, *pers. comm.*) reflecting the persistence of vector populations.

Malaria vectors are historically known to feed between dusk and dawn, with variations in activity time and biting rate among species [13]. In the present study, *An. funestus* s.s. biting was more intense in the second half of the night (24:00 p.m. to 05:00 p.m.), which is supported by similar findings reported in Senegal, Kenya, and Zambia [76,92,93]; hence, a regular and proper use of undamaged ITNs must be promoted in this region.

*Anopheles arabiensis* showed intense biting activity early in the first half of the night, which has also been observed in Ethiopia [75], while late peaks have been shown in Senegal and Uganda [76,94]. In a study performed in Tanzania, *An. arabiensis* showed late biting peaks indoors but early biting peak outdoors [95]. These variations in the night biting cycle may have serious implications on malaria transmission, as they highly complicate the deployment of vector control tools in space and time.

*Anopheles quadriannulatus*, the most abundant species collected, predominantly showed outdoor biting activity, which was more intense in the first half of the night. This is consistent with the previously described zoophilic and exophilic character [13]. In a study performed in south-east Zambia, *An. quadriannulatus* did not show a preference for indoor or outdoor biting but showed peak biting activity in the first half of the night, as confirmed by the finding in the present study [56]. Although this species’ biting activity was more intense early in the evening, the intensity of biting throughout of the rest of the night was considerably higher than that of the other two species.

*Plasmodium falciparum* sporozoite presence was detected in *An. funestus* s.s. and *An. arabiensis* in this study, and these two species are important vectors for transmission of malaria in the study village and globally. The overall SR of these two species was similar to those previously reported in Matola in southern Mozambique [50]. Although the SRs were comparable, the EIRs estimated for these regions were different, being three times higher in Matola than in the study area [50]. This difference in the EIR may reflect variations in the biting rates of these mosquito species [96]. Both SRs and EIRs in this study were relatively lower than those reported in Kenya and Tanzania [87,97]. The EIR is a measure of transmission intensity and may influence the morbidity of *Plasmodium falciparum* cases [50]. Beier (1999) reported a relationship between the EIR and the prevalence of malaria in Africa, where an increase in the EIR was related to an increase in malaria prevalence [98]. The EIR in the present study was relatively low and could be related to the malaria prevalence of 11.9% found in 2019 (Salome, *unpublished data*). However, high prevalence rates of malaria in areas with extremely low levels of EIR have been reported elsewhere in Africa [99,100], suggesting that other factors may influence malaria transmission [101]. All positive specimens were collected outdoors, suggesting that the transmission of malaria may be mostly occurring outdoors. This finding may have implications in malaria control in the study region, as the main vector control intervention deployed to target vectors is only applied indoors.

*Anopheles quadriannulatus* is considered a non-vector species because of its preference to feed on animals [74]; however, the susceptibility of *An. quadriannulatus* to infection by *P. falciparum* was demonstrated in the laboratory [102]. Supporting this species ability to vector the *Plasmodium* parasite, a previous study by Lobo et al. reported the presence of infected *An. quadriannulatus* only with PCR analysis [23]. Such findings of *Plasmodium*-infected *An. quadriannulatus*, aligned with the fact that a relatively high proportion of this mosquito species was collected while trying to feed on humans during this study, can be considered to support its potential as a malaria vector. However, the lower susceptibility to infection by *P. falciparum* in the laboratory, compared with that of *An. gambiae* s.s. and *An. stephensi* mediated by immunologic resistance [103], and the zoophilic character of *An. quadriannulatus* [13] may ultimately still contribute to it being considered a species of low medical importance [74].

Some limitations of the study included the collection period not spanning the full period of two consecutive seasons. It covered five months of the dry season and four months of the wet season. Although most of the two seasons were covered, a full period (12 months) of collection could allow a better analysis of the patterns of distribution of mosquitoes by season. Moreover, only one collection method was used. Human landing catches do not provide high yields of host-seeking mosquitoes, as it depends on the collector attractiveness and skills. A better yield could be achieved with the use of complementary light-trap collections that can be deployed in much higher quantities per night and in multiple houses without the potential risk of collectors contracting vector-borne diseases [104]. Blood meal analysis to confirm the origin of blood was also not performed. These analyses could contribute to the knowledge of the host preferences of *An. quadriannulatus*, the most abundant species of *Anopheles* mosquitoes found in the study region.

## 5. Conclusions

Evidence of the diversity and abundance of host-seeking *Anopheles* mosquitoes from Conhane Village is reported for the first time. The significant proportion of *An. arabiensis* biting outdoors and infected mosquitoes biting early in the evening require the inclusion of additional measures to reduce outdoor biting and thus reduce transmission. Vector control interventions targeting endophagic vectors should continue to be used, and interventions to reduce outdoor exposure, such as topical and spatial repellents, should be considered in further research.

## Figures and Tables

**Table 1:** Genera composition, abundance, and distribution of collected mosquitoes by site.

Genus	Conhane N (%)	Cotsuane N (%)	Total N (%)
*Mansonia*	1202 (19.6)	4922 (80.4)	6124 (46.9)
*Culex*	1995 (55.1)	1624 (44.9)	3619 (27.7)
*Anopheles*	681 (37.7)	1124 (62.3)	1805 (13.8)
*Aedes*	42 (62.7)	25 (37.3)	67 (0.5)
All other genera	581 (40.2)	863 (59.8)	1444 (11.1)
Total	4501 (34.5)	8558 (65.5)	13,059 (100.0)

**Table 2:** Proportions of *An. funestus* s.s., *An. arabiensis*, and *An. quadriannulatus* by site.

*Anopheles* Species	Conhane N (%)	Cotsuane N (%)	Total N (%)
*funestus s.s.*	7 (2.5)	69 (9.6)	76 (7.6)
*arabiensis*	51 (18.0)	66 (9.1)	117 (11.6)
*quadriannulatus*	225 (79.5)	587 (81.3)	812 (80.8)
Total	283	722	1005

**Table 3:** Indoor and outdoor proportions of *An. funestus* s.s., *An. arabiensis*, and *An. quadriannulatus*.

*Anopheles* Species	Indoor N (%)	Outdoor N (%)	Total N (%)
*funestus s.s.*	33 (17.3)	43 (5.3)	76 (7.6)
*arabiensis*	25 (13.1)	92 (11.3)	117 (11.6)
*quadriannulatus*	133 (69.6)	679 (83.4)	812 (80.8)
Total	191	814	1005

## Data Availability

The data presented in this study are available upon request from the corresponding author.
